# Sector-dependent device discordance of RNFL measurements: A prospective cross-sectional study of cross-platform variability and interchangeability in glaucoma monitoring

**DOI:** 10.1097/MD.0000000000049704

**Published:** 2026-07-10

**Authors:** Fevzi Akkan, Ebru Görgün

**Affiliations:** a Department of Ophthalmology, Dunyagoz Eye Hospital, Istanbul, Türkiye.

**Keywords:** device-specific bias, glaucoma progression monitoring, optical coherence tomography, RNFL interchangeability, sector-dependent device discordance

## Abstract

This study aimed to characterize intra-device repeatability and sector-dependent inter-device variability of peripapillary retinal nerve fiber layer (RNFL) measurements across 5 optical coherence tomography (OCT) platforms and define the concept of sector-dependent device discordance, the non-uniform distribution of inter-device measurement bias across anatomical RNFL sectors. This prospective cross-sectional study included 38 healthy participants who underwent peripapillary RNFL imaging using 5 OCT devices: Heidelberg Spectralis, TowardPi BMIZAR 400 kHz swept-source OCT, Huvitz HOCT-1/1F, NIDEK RS-1 Glauvas, and Topcon Maestro 2. RNFL thickness was analyzed in the superior, nasal, inferior, and temporal sectors, as well as global mean thickness. Three consecutive measurements were obtained for each eye with each device to assess intra-device repeatability. Repeatability was evaluated using repeated-measures analysis of variance. Inter-device reliability was assessed using intraclass correlation coefficients, with Heidelberg Spectralis as the reference. Agreement between devices was further evaluated using Bland–Altman analysis. Excellent intra-device repeatability was documented across all platforms. However, marked inter-device variability demonstrated sector-dependent patterns: global RNFL measurements showed moderate agreement (intraclass correlation coefficient: 0.346–0.805), while nasal and temporal sectors exhibited disproportionately higher variability (absolute percentage error up to 20.76% in the nasal sector; limits of agreement width ranging from 20.23% to 35.52% in temporal). Bland–Altman analyses revealed device-specific bias signatures (Huvitz HOCT-1/1F: −8.17% global bias; NIDEK RS-1 Glauvas: +10.59%; Topcon Maestro 2: −4.47%), indicating systematic, reproducible platform-inherent differences rather than random measurement noise. While intra-device repeatability is excellent, inter-device RNFL measurements remain substantially discordant, particularly in nasal and temporal sectors, where absolute percentage error reaches up to 20.76% and limits of agreement exceed 35%, a phenomenon termed sector-dependent device discordance. Global RNFL measurements may appear concordant while sectoral measurements diverge, creating a theoretically plausible risk for false progression detection, pending validation in glaucomatous eyes. RNFL measurements are not interchangeable across platforms and are particularly unreliable for sector-specific assessment. Device-consistent monitoring is essential for glaucoma follow-up; multicenter studies and device switching scenarios warrant platform-specific normalization strategies.

## 1. Introduction

Peripapillary retinal nerve fiber layer (RNFL) thickness measurements obtained by optical coherence tomography have become the cornerstone of glaucoma diagnosis and monitoring. However, a critical yet underappreciated challenge persists: RNFL measurements are not only device-dependent but also sector-dependent, with systematic variability that differs fundamentally across anatomical regions of the optic nerve head. In particular, RNFL thickness measurements are widely used in clinical practice and research as quantitative markers of axonal integrity, supporting the evaluation of optic nerve health and longitudinal structural change.^[1,2]^ In healthy individuals, RNFL measurements also form the basis of normative databases that underpin diagnostic thresholds and progression analyses applied in clinical settings.

An emerging paradigm in optical coherence tomography (OCT)-based glaucoma monitoring recognizes that RNFL measurements exhibit sector-dependent device variability distinct from global measurement patterns. While inter-device differences in global RNFL thickness have been described, their non-uniform distribution across anatomical sectors has not previously been defined as a unified construct. We term this pattern sector-dependent device discordance (SDDD). Although sector-specific variability has been noted in prior 2-device comparisons,^[3]^ it has not been formally characterized as a coherent framework. SDDD creates a potentially clinically relevant scenario in which cross-device measurement concordance at the global level may obscure substantial sectoral divergence. This sector-dependent variability may theoretically introduce a risk of false disease progression detection in clinical scenarios involving multi-platform longitudinal imaging, though this inference requires validation in disease populations.

In daily practice, clinicians frequently interpret RNFL thickness values using absolute measurements, sectoral profiles, and interocular comparisons. However, RNFL measurements are known to be influenced by device-specific factors, including optical design, scan acquisition protocols, reference circle geometry, and proprietary automated segmentation algorithms. Consequently, RNFL thickness values obtained from different OCT platforms may not be directly interchangeable, even when measurements are acquired in the same eye under standardized conditions.^[4–7]^ This issue is particularly relevant in healthy and normative cohorts, where small systematic differences between devices may be misinterpreted as physiological variability rather than measurement-related bias.

While previous studies have established that individual OCT platforms demonstrate excellent intra-device repeatability, a paradoxical discrepancy exists: high repeatability within a single device does not ensure cross-platform interchangeability. More importantly, this inter-device variability is not uniformly distributed across RNFL sectors; nasal and temporal regions exhibit disproportionately larger measurement divergence than global measurements would suggest.^[8,9]^ Nevertheless, inter-device agreement has been reported to be variable, with Bland–Altman analyses and intraclass correlation coefficients (ICCs) often revealing clinically relevant systematic differences between instruments.^[5,10]^ Similar findings have also been reported in studies including glaucoma, ocular hypertension, or other optic nerve pathologies, underscoring that measurement variability across OCT platforms persists across both healthy and diseased eyes and may influence clinical interpretation and research comparability.^[11–14]^

From a methodological and clinical perspective, it is therefore crucial to characterize both intra-device consistency and inter-device comparability of RNFL thickness measurements in healthy subjects. Establishing the extent of agreement and systematic bias between different OCT systems in a normative population provides an essential reference framework for interpreting RNFL measurements in routine practice and multicenter research. Accordingly, the present study aimed to comprehensively characterize the sector-dependent nature of inter-deviceRNFL variability and to formally define SDDD. Specific objectives were: to quantify intra-device repeatability across all 5 OCT platforms; to characterize inter-device agreement patterns stratified by anatomical sector; to identify device-specific RNFL measurement bias signatures; and to elucidate the clinical implications of sector-dependent variability for longitudinal glaucoma monitoring.

The concept of SDDD is a clinically relevant observation: RNFL measurements from different OCT systems may agree well at the global level while diverging substantially in specific sectors. This sector-dependent nature is particularly concerning for glaucoma follow-up, where focal RNFL thinning in vulnerable sectors (superior and inferior) is the hallmark of structural progression. False progression detection, where apparent RNFL loss reflects device-switching artifact rather than true optic nerve degeneration, represents a plausible clinical risk that warrants systematic characterization, beginning with a normative reference framework as established in the present study.

## 2. Materials and methods

### 2.1. Study design and participants

This prospective, cross-sectional study was approved by the Medipol University Institutional Ethics Committee (Approval number: 1647). All procedures were conducted in accordance with the principles of the Declaration of Helsinki. Healthy volunteers were consecutively recruited from the outpatient ophthalmology clinic. Written informed consent was obtained from all participants before enrollment.

**Figure 1.**
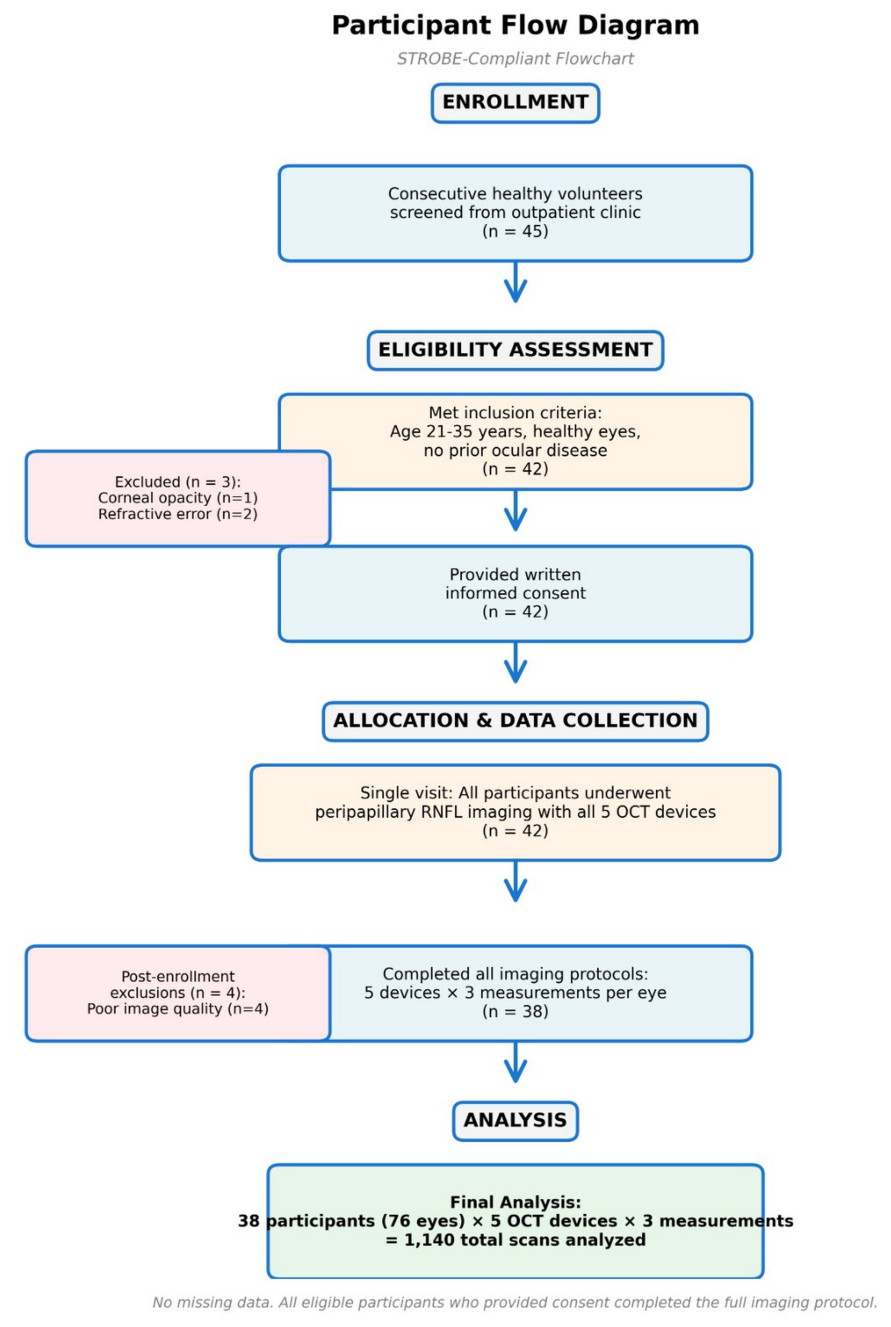
Flow diagram of participant selection and study design.

Subjects with a history of ocular disease, glaucoma or glaucoma suspicion, previous ocular surgery, optic nerve pathology, significant refractive error (>±3.0 diopters spherical equivalent), media opacities affecting image quality, systemic diseases known to affect the optic nerve or retina, or poor fixation were excluded from the study. The participant selection process is illustrated in Figure 1.

### 2.2. Ophthalmic examination

All participants underwent a comprehensive ophthalmic examination including best-corrected visual acuity assessment, intraocular pressure measurement, slit-lamp biomicroscopy, and dilated fundus examination. Only eyes with normal ophthalmic findings were included in the final analysis.

### 2.3. OCT imaging protocol

Peripapillary RNFL thickness measurements were obtained using 5 different OCT devices: Heidelberg Spectralis OCT (HRA), TowardPi BMIZAR 400 kHz ultra-widefield full-range swept-source optical coherence tomography (BMIZAR), Huvitz HOCT-1/1F (HUVITZ), NIDEK RS-1 Glauvas (NIDEK), and Topcon Maestro 2 (TOPCON). To minimize operator- and time-related variability, all OCT examinations were performed during the same visit on the same day under identical lighting conditions. Each eye was examined sequentially with all 5 devices, and all scans were acquired by the same experienced operator.

For each device, peripapillary RNFL imaging was performed using the manufacturer’s recommended optic disc-centered circular scan protocol. RNFL thickness values were automatically calculated by the proprietary software of each device without manual correction in order to reflect real-world clinical practice. RNFL thickness was analyzed for the superior, nasal, inferior, and temporal sectors, as well as the global mean RNFL thickness. Scans with poor image quality, motion artifacts, segmentation errors, or inadequate centration were excluded from the analysis.

### 2.4. Device characteristics

The Huvitz HOCT-1/1F is an all-in-one Spectral-Domain OCT system operating at approximately 840 nm. Depth-resolved retinal reflectivity is obtained through Fourier transformation of the interference spectrum. Peripapillary RNFL thickness values are derived using automated segmentation algorithms identifying the RNFL boundaries.

The NIDEK RS-1 Glauvas is a Spectral-Domain OCT device utilizing high-speed A-scan acquisition and proprietary segmentation algorithms. RNFL thickness is defined based on automated delineation of the inner limiting membrane and the outer RNFL boundary along the peripapillary scan circle.

Topcon Maestro 2 is an automated Spectral-Domain OCT system integrated with a true-color fundus camera. Automated alignment, autofocus, and image acquisition enable consistent optic disc-centered scanning, and RNFL thickness maps are generated using built-in software without operator-dependent adjustments.

The TowardPi BMIZAR 400 kHz system is an ultra-widefield Swept-Source OCT platform operating at high scan speeds. Swept-source technology employs a rapidly tunable laser to achieve deeper tissue penetration and wide-field imaging.^[15]^ Peripapillary RNFL measurements are obtained from high-density circular scans centered on the optic nerve head using automated segmentation.

The Heidelberg Spectralis OCT combines Spectral-Domain OCT with confocal scanning laser ophthalmoscopy. Active eye-tracking technology ensures precise scan registration and repeatable acquisition. RNFL thickness measurements are obtained using device-specific automated segmentation algorithms. The Heidelberg Spectralis was selected as the reference device for inter-device comparisons for several reasons. First, Spectralis is among the most extensively validated OCT platforms in the published literature, with a large body of normative data and reproducibility studies supporting its use as a benchmark instrument in RNFL research.^[2,9,16]^ Second, the active eye-tracking (TruTrack) technology of Spectralis enables highly precise scan registration and repositioning, which minimizes scan-to-scan positional variability and is recognized as a technical advantage for reproducibility in peripapillary imaging. Third, Spectralis-derived RNFL measurements have been widely used as reference values in prior cross-platform comparison studies, facilitating contextual comparison of our findings with the existing literature.^[5,17]^ Finally, from a practical standpoint, Spectralis demonstrated the lowest global coefficient of variation (CV%) (0.42%) among all 5 devices in this study, confirming its superior within-session measurement consistency and reinforcing its suitability as the reference standard. It should be noted that the designation of Spectralis as the reference reflects its role as an analytical anchor for bias quantification rather than a claim that it represents the absolute ground truth for RNFL thickness measurement, given that no such external gold standard currently exists.

### 2.5. Repeatability assessment

To assess intra-device repeatability, 3 consecutive peripapillary RNFL measurements were obtained from each eye for each OCT device during the same visit. Participants were asked to reposition their head between acquisitions to minimize positional bias. Right and left eyes were analyzed separately, as interocular asymmetry in RNFL measurements has been documented in normal individuals,^[18]^ and repeated measurements were labeled as 3 repeated right eye measurements (R1–R3) and 3 repeated left eye measurements (L1–L3), respectively. All measurements across all 5 devices were performed by the same experienced operator in a single session, thereby eliminating interobserver variability by design. The 3 consecutive measurements obtained per eye per device also served as a functional assessment of intraobserver repeatability, as all acquisitions were performed by the same operator under identical conditions. Intraobserver consistency was quantified using the CV% calculated from the 3 repeated measurements per eye per device, and within-session repeatability was further evaluated using repeated-measures analysis of variance (ANOVA) as described in the Section 2.6.

### 2.6. Statistical analysis

Peripapillary RNFL thickness measurements were obtained using 5 different imaging devices (HRA, BMIZAR, HUVITZ, NIDEK, and TOPCON). Descriptive statistics were expressed as mean ± standard deviation. Normality of continuous variables was assessed using the Shapiro–Wilk test. To evaluate intra-device repeatability across 3 consecutive measurements obtained from the same eye (R1–R3 and L1–L3), repeated measures ANOVA was performed separately for each device and RNFL sector (superior, nasal, inferior, temporal, and global mean). When a statistically significant overall effect was detected, pairwise comparisons were conducted using Bonferferroni-adjusted post hoc tests. As an index of intraobserver measurement consistency, the within-subject CV% was calculated for each device and RNFL sector from the 3 repeated measurements per eye, providing a standardized metric of operator-level repeatability independent of between-device differences. Differences between right and left eyes were analyzed using paired samples *t* tests with bootstrap resampling to account for potential deviations from normality. Inter-device reliability between the reference method (HRA, Heidelberg Spectralis) and each of the other devices was assessed using the ICC. The selection of HRA as the reference is justified in the Section 2.4 and reflects established precedent in the OCT comparative literature rather than an assumption of absolute measurement superiority. A 2-way random-effects model with absolute agreement was applied [ICC (2,1)], and ICC values were reported together with their 95% confidence intervals. ICC values were interpreted according to commonly accepted thresholds, with higher values indicating better inter-method reliability. Agreement between devices was further evaluated using Bland–Altman analysis, following established reporting standards,^[19]^ with mean bias expressed as percentage difference relative to the reference method. Limits of agreement (LoA) were calculated as the mean difference ± 1.96 standard deviation, and the width of the LoA was reported as a measure of agreement dispersion. Proportional bias was assessed using linear regression analysis of the differences against the mean values, with the significance of the regression slope used to identify proportional bias. In addition, absolute percentage error (APE) was calculated to quantify relative measurement error between devices. All statistical analyses were performed using IBM Statistical Package for the Social Sciences Statistics (IBM Corporation, Armonk, NY) and MedCalc 14 (Acacialaan 22, B-8400 Ostend, Belgium) Statistical Software. No formal a priori sample size calculation was performed for inter-device agreement analyses, as this study was primarily designed as a comprehensive observational characterization study; accordingly, the precision of agreement estimates should be interpreted within the context of the sample size as discussed in the Section 6.

#### 2.6.1. Sector-dependent bias analysis

To formally assess Sector-Dependent Device Discordance (SDDD), proportional bias analysis and APE were stratified by anatomical RNFL sector (superior, nasal, inferior, temporal) separately from global measurements. This sector-specific stratification approach allowed identification of differential measurement patterns across regions and quantification of relative error in clinically vulnerable nasal and temporal sectors versus global assessment. This methodological approach specifically addresses whether inter-device agreement patterns differ systematically by anatomical region, which is a key feature distinguishing this study from prior 2-device comparisons and enabling formal characterization of SDDD phenomena.

## 3. Results

A total of 38 participants were included in the study, with a mean age of 26.6 ± 3.4 years and a median age of 26 years (range: 21–35). Of these, 26 participants (68.4%) were female, and 12 (31.6%) were male.

### 3.1. Global RNFL agreement versus sector-dependent variability

RNFL thickness measurements obtained using 5 different devices (HRA, BMIZAR, HUVITZ, NIDEK, and TOPCON) showed marked inter-device variability. Notably, global mean RNFL thickness demonstrated moderate agreement between selected device pairs (e.g., HRA-BMIZAR ICC = 0.805), while sectoral measurements, particularly nasal and temporal sectors, exhibited disproportionately higher variability and wider LoA. This pattern exemplifies the concept of SDDD, wherein device-specific bias is not uniformly distributed across anatomical regions. The results are summarized in the tables below.

RNFL thickness measurements acquired with the 5 devices were compared across the superior, nasal, inferior, temporal, and global mean sectors. Overall, statistically significant differences in RNFL thickness were observed among the devices in all sectors (repeated-measures ANOVA, *P* < .001 for all sectors).

In the superior RNFL sector, when right and left eye averages were analyzed together, the HUVITZ device yielded the highest measurements (142.00 ± 14.10 µm), whereas the NIDEK device showed the lowest values (120.36 ± 10.30 µm). Differences between HRA and BMIZAR, HUVITZ, and NIDEK were statistically significant (all *P* < .001), while no significant difference was observed between HRA and TOPCON (*P* = .999). In right–left eye comparisons, significant asymmetry was observed with the HRA (*P* = .010) and TOPCON (*P* = .036) devices, whereas no significant right–left difference was detected with the other devices.

In the nasal RNFL sector, the HUVITZ device provided the highest mean thickness (96.93 ± 19.94 µm), while the NIDEK device produced the lowest measurements (71.00 ± 12.52 µm). Comparisons between HRA and all other devices revealed statistically significant differences (most comparisons *P* < .001). In right–left eye comparisons, significant differences were found for the BMIZAR (*P* = .001) and HUVITZ (*P* = .015) devices, whereas measurements obtained with HRA, NIDEK, and TOPCON were similar between eyes.

In the inferior RNFL sector, marked differences among devices were observed. The highest mean RNFL thickness was measured with the BMIZAR device (132.25 ± 12.72 µm), whereas the lowest values were obtained with the NIDEK device (91.34 ± 9.85 µm). Differences between HRA and all other devices were statistically significant (all *P* < .001). In right–left eye comparisons, a significant difference was detected only for the TOPCON device (*P* = .011), while no significant asymmetry was observed for the remaining devices.

In the temporal RNFL sector, the highest mean values were obtained with the HUVITZ device (81.39 ± 9.26 µm), and the lowest values with the NIDEK device (61.38 ± 8.96 µm). Differences between HRA and HUVITZ, NIDEK, and TOPCON were statistically significant (all *P* < .001), whereas no significant difference was found between HRA and BMIZAR (*P* = .236). Significant right–left asymmetry was observed for the HRA, NIDEK, and TOPCON devices (all *P* ≤ .001), while no significant asymmetry was detected for BMIZAR and HUVITZ.

When global mean RNFL thickness was evaluated, the HUVITZ device yielded the highest mean value (113.24 ± 9.90 µm), whereas the NIDEK device showed the lowest global RNFL thickness (93.94 ± 8.68 µm). Differences between HRA and BMIZAR (*P* = .042), as well as between HRA and HUVITZ, NIDEK, and TOPCON, were statistically significant (all other comparisons *P* < .001). In right–left eye comparisons, a significant difference was observed only for the BMIZAR device (*P* = .003) (Table 1).

**Table 1:** Comparison of retinal nerve fiber layer (RNFL) thickness measurements across 5 optical coherence tomography devices.

(n = 38)	I	II	III	IV	V	*P* value*	Pairwise comparisons			
	HRA	BMIZAR	HUVITZ	NIDEK	TOPCON					
	Mean (SD)	Mean (SD)	Mean (SD)	Mean (SD)	Mean (SD)		I vs II	I vs III	I vs IV	I vs V
RNFL thickness (µm)										
Superior										
Mean of right and left eyes (µm)	131.96 (10.18)	125.36 (8.82)	142.00 (14.10)	120.36 (10.30)	133.24 (11.09)	<.001	<0.001	<0.001	<0.001	0.999
Right eye mean (Rep 1–3) (µm)	130.00 (10.46)	125.59 (9.06)	141.39 (11.37)	119.39 (10.65)	131.43 (11.98)	<.001	0.075	<0.001	<0.001	0.999
Left eye mean (Rep 1–3) (µm)	133.91 (11.94)	125.13 (9.70)	142.61 (19.77)	121.32 (11.14)	135.04 (12.14)	<.001	<0.001	0.027	<0.001	0.999
*P* value (right vs left)†	.010	.659	.653	.094	.036					
Nasal										
Mean of right and left eyes (µm)	76.44 (14.68)	84.34 (8.39)	96.93 (19.94)	71.00 (12.52)	85.67 (11.50)	<.001	<0.001	<0.001	0.030	<0.001
Right eye mean (Rep 1–3) (µm)	77.60 (14.49)	86.68 (8.27)	95.46 (11.57)	70.98 (12.50)	86.11 (12.32)	<.001	<0.001	<0.001	0.002	<0.001
Left eye mean (Rep 1–3) (µm)	77.37 (12.58)	82.00 (9.61)	92.23 (12.03)	71.02 (13.91)	85.23 (12.10)	<.001	0.061	<0.001	<0.001	<0.001
*P* value (right vs left)†	.852	.001	.015	.981	.531					
Inferior										
Mean of right and left eyes (µm)	102.30 (10.17)	132.25 (12.72)	110.20 (8.47)	91.34 (9.85)	107.04 (8.57)	<.001	<0.001	<0.001	<0.001	<0.001
Right eye mean (Rep 1–3) (µm)	132.69 (19.34)	132.25 (14.37)	139.00 (13.96)	121.31 (16.49)	138.18 (17.08)	<.001	0.999	0.035	<0.001	0.001
Left eye mean (Rep 1–3) (µm)	134.72 (15.23)	132.25 (12.54)	135.78 (18.52)	123.39 (16.90)	142.16 (14.65)	<.001	0.999	0.999	<0.001	<0.001
*P* value (right vs left)†	.368	.999	.172	.223	.011					
Temporal										
Mean of right and left eyes (µm)	71.91 (9.26)	69.75 (6.52)	81.39 (9.26)	61.38 (8.96)	75.89 (8.13)	<.001	0.236	<0.001	<0.001	<0.001
Right eye mean (Rep 1–3) (µm)	74.61 (10.85)	70.57 (7.33)	82.87 (10.09)	63.65 (9.75)	78.35 (8.28)	<.001	0.029	<0.001	<0.001	<0.001
Left eye mean (Rep 1–3) (µm)	69.01 (7.98)	68.94 (6.61)	80.17 (10.70)	59.30 (8.66)	73.88 (8.12)	<.001	0.999	<0.001	<0.001	<0.001
*P* value (right vs left)†	.001	.052	.100	.001	.001					
Mean										
Mean of right and left eyes (µm)	103.47 (8.29)	101.44 (5.78)	113.24 (9.90)	93.94 (8.68)	108.40 (7.81)	<.001	0.042	<0.001	<0.001	<0.001
Right eye mean (Rep 1–3) (µm)	103.52 (7.85)	102.38 (6.17)	113.77 (9.31)	93.87 (8.77)	107.70 (8.74)	<.001	0.764	<0.001	<0.001	0.004
Left eye mean (Rep 1–3) (µm)	103.42 (9.28)	100.50 (5.83)	112.70 (11.98)	94.01 (8.88)	109.10 (8.27)	<.001	0.020	<0.001	<0.001	<0.001
*P* value (right vs left)†	.905	.003	.469	.765	.257					

R1–R3 and L1–L3 represent 3 repeated measurements obtained from the right and left eyes, respectively.

BMIZAR = TowardPi BMIZAR 400 kHz SS-OCTA, HRA = Heidelberg Spectralis OCT, HUVITZ = Huvitz HOCT-1/1F, NIDEK = NIDEK RS-1 Glauvas, RNFL = retinal nerve fiber layer, SS-OCTA = swept-source optical coherence tomography, SD = standard deviation, TOPCON = Topcon Maestro 2.

* Repeated measures analysis of variance (ANOVA) was performed to evaluate the differences in measurement results between 5 devices in the same patients, Adjustment for multiple comparisons: Bonferroni.

† Paired samples *t* test (Bootsrap).

Within-device repeatability of RNFL thickness measurements was evaluated using 3 consecutive acquisitions obtained from the right and left eyes (R1–R3 and L1–L3). In the superior RNFL segment, no statistically significant differences were observed among the 3 consecutive measurements for either eye across all devices (all *P* > .05), indicating high short-term repeatability in this segment. As a complementary metric of intraobserver repeatability, the within-subject CV% was calculated across the 3 consecutive measurements for each eye and device. Global mean RNFL CV% was low across all platforms (overall mean 1.65%), with device-specific global CV% of 0.42% for HRA (Spectralis), 0.52% for NIDEK, 1.51% for BMIZAR, 2.47% for Topcon, and 3.33% for Huvitz. Sectoral CV% values were generally higher than global values (particularly in nasal and temporal sectors) with nasal CV% ranging from 2.11% (HRA) to 6.57% (Huvitz), and temporal CV% ranging from 1.04% (HRA) to 5.01% (Huvitz). Since all measurements were performed by a single operator, interobserver variability could not be formally assessed; however, the elimination of inter-operator differences through this single-operator design represents a methodological strength that isolates device-related variability from operator-related variability.

In the nasal RNFL segment, no significant differences were observed among consecutive measurements in the right eye for any device (all *P* > .05). In the left eye, a statistically significant overall effect was detected only for the NIDEK device (*P* = .025). Post hoc analysis in the NIDEK group revealed that this difference was primarily driven by the comparison between the L1 and L2 measurements (*P* = .043), whereas comparisons between L1–L3 (*P* = .174) and L2–L3 (*P* = .999) were not significant. After Bonferroni correction for multiple comparisons, this finding was considered borderline and interpreted with caution.

In the inferior RNFL segment, no statistically significant differences were detected among the 3 consecutive measurements for either eye across all devices (all *P* > .05).

In the temporal RNFL segment, a statistically significant difference among consecutive measurements was observed only for the NIDEK device in the right eye (*P* = .021), while no significant differences were detected for the other devices. In the left eye, no significant differences were observed among the 3 measurements for any device (all *P* > .05). Although 1 pairwise comparison in the NIDEK group showed borderline significance for the left eye (*P* = .040), this finding was considered exploratory given the nonsignificant overall test.

For global mean RNFL thickness, no statistically significant differences were observed among the 3 consecutive measurements in either eye across all devices (all *P* > .05) (Table 2).

**Table 2:** Within-device repeatability of retinal nerve fiber layer (RNFL) thickness measurements across 3 consecutive acquisitions (R1–R3 and L1–L3) obtained with 5 optical coherence tomography devices.

	HRA	BMIZAR	HUVITZ	NIDEK	TOPCON
	Mean (SD)	Mean (SD)	Mean (SD)	Mean (SD)	Mean (SD)
RNFL thickness (µm)					
Superior					
R1	129.82 (10.11)	125.37 (10.11)	141.05 (14.19)	119.55 (10.56)	133.00 (10.24)
R2	130.21 (10.53)	126.21 (9.43)	142.45 (12.31)	119.42 (11.06)	127.92 (22.88)
R3	129.97 (10.83)	125.18 (9.51)	140.68 (12.78)	119.21 (10.51)	133.37 (11.78)
*P* value (R1 vs R2 vs R3)	.263	.499	.602	.571	.155
L1	133.63 (11.96)	125.08 (9.98)	142.82 (19.66)	121.45 (11.27)	135.95 (12.67)
L2	133.82 (12.14)	125.42 (10.45)	142.08 (20.74)	121.05 (11.11)	135.53 (11.30)
L3	134.29 (11.95)	124.89 (10.39)	142.92 (21.20)	121.47 (11.17)	133.66 (14.34)
*P* value (L1 vs L2 vs L3)	.237	.794	.843	.261	.139
Nasal					
R1	77.58 (14.28)	86.08 (8.34)	95.74 (11.79)	70.47 (12.50)	87.24 (12.64)
R2	77.71 (14.64)	87.53 (9.04)	95.45 (13.59)	71.21 (12.17)	84.16 (15.52)
R3	77.50 (14.61)	86.45 (8.46)	95.21 (11.49)	71.26 (13.10)	86.92 (11.65)
*P* value (R1 vs R2 vs R3)	.584	.093	.907	.144	.092
L1	77.50 (12.93)	82.05 (10.21)	91.66 (11.96)	71.50 (14.16)*	85.92 (12.44)
L2	77.24 (12.39)	81.58 (9.76)	92.82 (13.93)	70.63 (13.86)	85.16 (13.07)
L3	77.37 (12.60)	82.37 (9.80)	92.24 (12.94)	70.92 (13.86)	84.61 (12.09)
*P* value (L1 vs L2 vs L3)	.645	.532	.943	.025	.366
Inferior					
R1	134.32 (26.06)	131.61 (14.49)	138.50 (14.91)	121.32 (16.58)	138.42 (16.66)
R2	131.95 (17.83)	132.71 (15.25)	138.74 (15.19)	121.16 (16.39)	137.24 (17.74)
R3	131.82 (17.57)	132.45 (14.97)	139.76 (13.97)	121.45 (16.61)	138.87 (18.13)
*P* value (R1 vs R2 vs R3)	.373	.585	.583	.658	.316
L1	134.92 (15.16)	132.76 (13.18)	135.53 (19.95)	121.55 (23.83)	141.66 (15.25)
L2	134.63 (15.06)	132.37 (13.36)	135.37 (19.28)	124.50 (15.20)	142.45 (14.33)
L3	134.61 (15.57)	131.63 (12.82)	136.45 (19.18)	124.13 (15.30)	142.37 (14.99)
*P* value (L1 vs L2 vs L3)	.471	.573	.789	.300	.475
Temporal					
R1	74.68 (10.91)	71.87 (11.08)	82.16 (11.44)	64.05 (9.80)†	77.71 (8.89)
R2	74.61 (10.80)	70.11 (6.90)	83.84 (12.21)	63.18 (9.89)	79.66 (11.18)
R3	74.53 (10.90)	69.74 (8.50)	82.61 (10.86)	63.71 (9.75)	77.68 (8.95)
*P* value (R1 vs R2 vs R3)	.662	.285	.537	.021	.281
L1	68.32 (7.73)	68.58 (7.10)	80.18 (12.26)	59.32 (8.56)	73.71 (8.61)
L2	69.42 (8.46)	69.29 (6.86)	80.13 (11.75)	59.53 (8.81)	73.82 (7.98)
L3	69.29 (8.36)	68.95 (7.25)	80.18 (10.78)	59.05 (8.73)	74.11 (8.28)
*P* value (L1 vs L2 vs L3)	.091	.604	.994	.187	.692
Mean					
R1	103.45 (7.59)	102.32 (6.20)	114.37 (8.15)	93.92 (8.75)	109.03 (7.53)
R2	103.58 (8.09)	102.74 (6.20)	112.42 (18.13)	93.76 (8.87)	104.82 (17.48)
R3	103.53 (7.91)	102.08 (6.48)	114.53 (8.13)	93.92 (8.75)	109.26 (8.10)
*P* value (R1 vs R2 vs R3)	.62	.167	.497	.478	.129
L1	103.34 (9.28)	100.50 (5.98)	112.55 (12.49)	94.16 (8.84)	109.32 (8.41)
L2	103.47 (9.28)	100.45 (6.15)	112.61 (12.79)	93.92 (9.01)	109.24 (8.11)
L3	103.45 (9.31)	100.55 (6.26)	112.95 (11.89)	93.95 (8.82)	108.74 (8.69)
*P* value (L1 vs L2 vs L3)	.49	.98	.891	.154	.322

Repeated measures analysis of variance (ANOVA) was performed to evaluate differences across the 3 consecutive measurements. Adjustment for multiple comparisons: Bonferroni.

BMIZAR = TowardPi BMIZAR 400 kHz SS-OCTA, HRA = Heidelberg Spectralis OCT, HUVITZ = Huvitz HOCT-1/1F, NIDEK = NIDEK RS-1 Glauvas, RNFL = retinal nerve fiber layer, SS-OCTA = swept-source optical coherence tomography, SD = standard deviation, TOPCON = Topcon Maestro 2.

* Statistical significance according to the L2 measurement.

† Statistical significance according to the R2 measurement, R1–R3 and L1–L3 represent 3 repeated measurements obtained from the right and left eyes, respectively.

Inter-device reliability of RNFL thickness measurements was assessed using the ICC [ICC(2,1)], with HRA defined as the reference method. In the superior RNFL segment, agreement between HRA and TOPCON was good for both combined right–left eye averages and separate right and left eye analyses (ICC = 0.744–0.795). In contrast, agreement between HRA and BMIZAR and HUVITZ was low to moderate (ICC approximately 0.36–0.51), while agreement between HRA and NIDEK was weak (ICC approximately 0.26–0.33), indicating marked systematic differences among devices in this segment.

In the nasal RNFL segment, agreement between HRA and NIDEK and TOPCON was moderate to good (ICC = 0.655–0.747). Agreement between HRA and BMIZAR was moderate (ICC approximately 0.50–0.57), whereas agreement between HRA and HUVITZ remained weak (ICC approximately 0.29–0.36), demonstrating variable inter-device reliability in nasal RNFL measurements.

In the inferior RNFL segment, good to excellent agreement was observed across all devices in right eye analyses (ICC = 0.626–0.871). Similar findings were observed in the left eye for HRA–BMIZAR, HRA–HUVITZ, and HRA–TOPCON comparisons. However, when right and left eye averages were analyzed together, agreement between HRA and BMIZAR was low (ICC = 0.161), suggesting that segment-specific variability may influence averaged measurements.

In the temporal RNFL segment, agreement between HRA and TOPCON was good to excellent across all analyses (ICC = 0.741–0.834). Agreement between HRA and BMIZAR was moderate to good (ICC = 0.595–0.780), whereas agreement with HUVITZ and NIDEK was generally weak to moderate.

For global mean RNFL thickness, agreement between HRA and BMIZAR was good in both right eye and combined right–left eye analyses (ICC = 0.805–0.843). Agreement between HRA and TOPCON was also generally good (ICC = 0.613–0.765), while agreement with HUVITZ and NIDEK was low to moderate. The consistently significant p-values (mostly *P* < .001) support the presence of statistically significant inter-device variability (Table 3).

**Table 3:** Inter-device reliability of retinal nerve fiber layer (RNFL) thickness measurements assessed using the intraclass correlation coefficient [ICC(2,1)] with HRA as the reference method.

	HRA vs BMIZAR		HRA vs HUVITZ		HRA vs NIDEK		HRA vs TOPCON	
	ICC (95% C.I.)	*P* value	ICC (95% C.I.)	*P* value	ICC (95% CI)	*P* value	ICC (95% C.I.)	*P* value
RNFL thickness (µm)								
Superior								
R & L means	0.510 (0.078/0.751)	<.001	0.440 (0.001/0.709)	<.001	0.260 (-0.080/0.553)	.004	0.795 (0.641/0.887)	<.001
Means of R	0.475 (0.181/0.689)	<.001	0.363 (-0.076/0.666)	<.001	0.326 (-0.061/0.617)	<.001	0.744 (0.562/0.858)	<.001
Means of L	0.491 (-0.003/0.755)	<.001	0.425 (0.117/0.656)	.001	0.269 (-0.074/0.559)	.004	0.765 (0.594/0.870)	<.001
Nasal								
R & L means	0.496 (0.099/0.733)	<.001	0.291 (-0.102/0.618)	<.001	0.655 (0.375/0.816)	<.001	0.664 (-0.005/0.875)	<.001
Means of R	0.544 (0.007/0.795)	<.001	0.321 (-0.103/0.660)	<.001	0.658 (0.295/0.832)	<.001	0.709 (0.035/0.895)	<.001
Means of L	0.572 (0.288/0.759)	<.001	0.360 (-0.102/0.687)	<.001	0.713 (0.314/0.870)	<.001	0.747 (-0.026/0.920)	<.001
Inferior								
R & L means	0.161 (-0.038/0.492)	<.001	0.495 (-0.028/0.765)	<.001	0.422 (-0.098/0.737)	<.001	0.790 (0.170/0.926)	<.001
Means of R	0.803 (0.652/0.893)	<.001	0.683 (0.417/0.832)	<.001	0.626 (0.113/0.835)	<.001	0.871 (0.620/0.945)	<.001
Means of L	0.749 (0.569/0.861)	<.001	0.746 (0.562/0.859)	<.001	0.554 (0.063/0.791)	<.001	0.855 (-0.016/0.962)	<.001
Temporal								
R & L means	0.732 (0.527/0.854)	<.001	0.449 (-0.090/0.754)	<.001	0.509 (-0.081/0.826)	<.001	0.834 (0.182/0.946)	<.001
Means of R	0.595 (0.299/0.777)	<.001	0.561 (-0.017/0.812)	<.001	0.557 (-0.079/0.852)	<.001	0.833 (0.430/0.935)	<.001
Means of L	0.780 (0.615/0.879)	<.001	0.382 (-0.102/0.708)	<.001	0.481 (-0.092/0.803)	<.001	0.741 (0.035/0.911)	<.001
Mean								
R & L means	0.805 (0.607/0.902)	<.001	0.381 (-0.086/0.689)	<.001	0.346 (-0.090/0.659)	<.001	0.740 (0.022/0.912)	<.001
Means of R	0.843 (0.716/0.916)	<.001	0.266 (-0.087/0.566)	.002	0.330 (-0.093/0.644)	<.001	0.613 (0.263/0.801)	<.001
Means of L	0.710 (0.438/0.851)	<.001	0.456 (-0.029/0.733)	<.001	0.373 (-0.076/0.676)	<.001	0.765 (-0.053/0.932)	<.001

Inter-method reliability was evaluated using the intraclass correlation coefficient (ICC), 2-way random-effects model with absolute agreement [ICC(2,1)].

APE = absolute percentage error, BMIZAR = TowardPi BMIZAR 400 kHz SS-OCTA, CI = confidence interval, HRA = Heidelberg Spectralis OCT, HUVITZ = Huvitz HOCT-1/1F, ICC = intraclass correlation coefficient, LoA = limits of agreement, NIDEK = NIDEK RS-1 Glauvas, RNFL = retinal nerve fiber layer, SS-OCTA = swept-source optical coherence tomography, TOPCON = Topcon Maestro 2.

Bland–Altman analyses performed with HRA as the reference method demonstrated marked systematic differences and limited agreement among devices in RNFL thickness measurements. In the superior RNFL segment, the BMIZAR and NIDEK devices exhibited positive mean bias relative to HRA (5.44% and 10.11%, respectively), whereas measurements obtained with HUVITZ and TOPCON showed negative bias. The near-zero mean bias (−0.72%) and relatively narrow LoA (width 24.24%) observed for TOPCON suggest closer agreement with HRA in this segment. In contrast, the wide LoA widths observed for HUVITZ (56.45%) and NIDEK (36.39%) limit clinical interchangeability at the individual measurement level.

In the nasal RNFL segment, all devices demonstrated substantial absolute bias and wide LoA compared with HRA. Notably, the pronounced negative mean bias observed with the HUVITZ device (−17.69%) and its high median APE (20.76%) indicate poor inter-device agreement in this segment. Although the NIDEK device exhibited positive bias (9.62%), its wide LoA (55.95%) similarly limited clinical interchangeability.

In the inferior RNFL segment, BMIZAR, HUVITZ, and TOPCON demonstrated negative systematic bias relative to HRA, whereas the NIDEK device showed positive mean bias (10.81%). Among these, TOPCON provided more consistent measurements, with narrower LoA (16.99%) and lower APE values.

In the temporal RNFL segment, the most pronounced positive bias was observed with the NIDEK device (17.51%), while HUVITZ and TOPCON demonstrated negative bias relative to HRA. The LoA width varied considerably across devices (range 20.23–35.52%), with TOPCON demonstrating the narrowest agreement and BMIZAR the widest, indicating device-dependent limits of agreement in temporal RNFL measurements.

For global mean RNFL thickness, the BMIZAR device demonstrated the lowest mean bias (1.93%) and the narrowest LoA (18.02%) compared with HRA. In contrast, HUVITZ and NIDEK showed marked negative and positive systematic bias, respectively (Table 4).

**Table 4:** Bland–Altman agreement analysis, proportional bias, and absolute percentage error for inter-device comparison of retinal nerve fiber layer (RNFL) thickness measurements using HRA as the reference method.

	Mean bias % (95% CI)	LoA width %	Proportional bias (*P*)	Median APE % (95% CI)
RNFL thickness (µm)				
Superior				
HRA vs BMIZAR	5.44 (4.06 to 6.81)	29.04	<.001	6.51 (4.94–7.70)
HRA vs HUVITZ	−6.01 (−8.68 to −3.34)	56.45	.135	8.53 (6.91–10.45)
HRA vs NIDEK	10.11 (8.39 to 11.83)	36.39	<.001	10.18 (8.75–11.65)
HRA vs TOPCON	−0.72 (−1.87 to 0.42)	24.24	.016	3.18 (2.53–3.89)
Nasal				
HRA vs BMIZAR	−8.73 (−10.99 to −6.46)	47.85	<.001	11.42 (10.33–13.11)
HRA vs HUVITZ	−17.69 (−19.86 to −15.53)	45.76	<.001	20.76 (18.63–22.49)
HRA vs NIDEK	9.62 (6.97 to 12.27)	55.95	.001	11.13 (9.43–13.41)
HRA vs TOPCON	−10.16 (−11.76 to −8.56)	33.78	<.001	10.90 (9.47–12.61)
Inferior				
HRA vs BMIZAR	−6.77 (−9.25 to −4.30)	52.3	<.001	7.32 (4.84–10.94)
HRA vs HUVITZ	−3.90 (−5.73 to −2.07)	38.69	<.001	6.92 (5.43–8.52)
HRA vs NIDEK	10.81 (8.79 to 12.83)	42.7	.202	10.16 (8.44–11.58)
HRA vs TOPCON	−4.61 (−5.42 to −3.81)	16.99	<.001	5.05 (4.53–6.42)
Temporal				
HRA vs BMIZAR	2.99 (1.31 to 4.67)	35.52	<.001	5.70 (4.37–7.57)
HRA vs HUVITZ	−11.51 (−13.07 to −9.95)	32.85	<.001	11.75 (8.83–13.90)
HRA vs NIDEK	17.51 (15.89 to 19.12)	34.1	.425	15.55 (13.81–16.87)
HRA vs TOPCON	−5.65 (−6.61 to −4.69)	20.23	<.001	5.87 (4.73–7.04)
Mean				
HRA vs BMIZAR	1.93 (1.08 to 2.78)	18.02	<.001	2.65 (2.07–3.11)
HRA vs HUVITZ	−8.17 (−9.75 to −6.58)	33.5	.001	9.81 (8.49–11.25)
HRA vs NIDEK	10.59 (8.98 to 12.20)	33.95	<.001	10.17 (9.08–11.51)
HRA vs TOPCON	−4.47 (−5.45 to −3.50)	20.64	<.001	5.42 (4.59–6.02)

Agreement between methods was evaluated using Bland–Altman analysis, linear regression for proportional bias, and absolute percentage error (APE) analysis.

Mean bias is expressed as percentage difference relative to the reference method, LoA width represents the difference between the upper and lower limits of agreement derived from Bland–Altman analysis, proportional bias *P*-values were obtained from regression slope estimates.

APE = absolute percentage error, BMIZAR = TowardPi BMIZAR 400 kHz SS-OCTA, CI = confidence interval, HRA = Heidelberg Spectralis OCT, HUVITZ = Huvitz HOCT-1/1F, ICC = intraclass correlation coefficient, LoA = limits of agreement, NIDEK = NIDEK RS-1 Glauvas, RNFL = retinal nerve fiber layer, SS-OCTA = swept-source optical coherence tomography, TOPCON = Topcon Maestro 2.

Representative Bland–Altman percent-difference plots for global and nasal-sector measurements are provided in the Supplement (Supplemental Figures 1–2), with Supplemental Figure 1, Supplemental Digital Content 1 illustrating global mean RNFL agreement patterns across all device pairs, and Supplemental Figure 2, Supplemental Digital Content 2 highlighting the pronounced nasal-sector variability characteristic of SDDD. An ICC summary heatmap across all sectors and device pairs is provided in Supplemental Figure 3, Supplemental Digital Content 3.

### 3.2. SDDD: differential inter-device agreement across anatomical regions

Notable sector-dependent patterns emerged in inter-deviceagreement. While global mean RNFL showed moderate reliability between HRA and BMIZAR (ICC = 0.805), agreement for the inferior sector combined right-left analysis was substantially reduced (ICC = 0.161), suggesting that sector-specific variability dominates when bilateral measurements are aggregated. More striking was the differential performance of each device pair across sectors: HRA–TOPCON demonstrated good agreement in superior (ICC = 0.795) and temporal sectors (ICC = 0.834), yet moderate agreement in nasal (ICC = 0.664). In contrast, HRA–HUVITZ consistently showed poor agreement across all sectors (ICC range 0.29–0.49), with particularly pronounced mean bias in the nasal region (−17.69%, median APE 20.76%) and temporal region (−11.51%, APE 11.75%).

## 4. Discussion

In this study, we comprehensively evaluated intra-device repeatability and inter-device agreement of peripapillary RNFL thickness measurements obtained using 5 different OCT systems in a healthy population. The principal findings demonstrate that, while all devices provided excellent short-term repeatability for global and sectoral RNFL measurements, marked and statistically significant differences were observed between devices across all RNFL sectors. Inter-device reliability varied considerably depending on both the OCT platform and the anatomical sector analyzed, with the highest agreement generally observed between Heidelberg Spectralis and Topcon Maestro 2, and substantially lower agreement noted for other device comparisons. Bland–Altman analyses further revealed clinically relevant systematic bias and wide LoA between devices, indicating that RNFL measurements are not directly interchangeable across OCT systems, even under standardized acquisition conditions in healthy eyes.

### 4.1. SDDD: a distinct clinical challenge

Beyond the general observation that RNFL measurements are device-dependent, the present study formally characterizes and systematically quantifies a more nuanced phenomenon that has been inconsistently described in prior literature. While sector-specific OCT measurement variability has been noted in previous cross-device studies, these observations have typically been secondary findings in 2-device comparisons without formal quantitative characterization across 5 platforms simultaneously. The concept of SDDD, as operationalized here, synthesizes and extends these prior observations into a coherent framework, representing a methodological contribution to the systematic understanding of multi-platform RNFL measurement variability. While global mean RNFL thickness demonstrated moderate-to-good agreement between selected device pairs (e.g., HRA–BMIZAR ICC = 0.805), the nasal and temporal sectors exhibited consistently wider LoA and substantially higher APEs (nasal APE up to 20.76% for HRA–HUVITZ; temporal APE up to 15.55% for HRA–NIDEK).

This sector-dependent vulnerability creates a theoretically plausible clinical scenario: in a glaucoma patient monitored longitudinally across different devices, structural change apparent in the nasal or temporal quadrant with Device A may appear stable, or paradoxically suggest regression, when follow-up imaging is performed with Device B. Whether this occurs in practice, and at what frequency, requires prospective validation in glaucomatous eyes. The mechanisms underlying SDDD likely involve multiple interacting factors: sector-specific segmentation challenges such as vascular shadowing and thinner peripheral RNFL architecture, non-uniform scan circle geometry across devices, and algorithm-specific sensitivity profiles. Importantly, nasal and temporal sectors are precisely those most affected by hemispherical optic nerve vulnerability patterns in early glaucomatous disease, elevating the clinical stakes of this sector-dependent variability.

### 4.2. Device-specific RNFL signatures

Bland–Altman analyses revealed that each OCT platform demonstrated a characteristic bias profile relative to the reference device, suggesting not random measurement noise but rather systematic, platform-inherent differences:

HUVITZ consistently demonstrated negative bias (mean global bias: −8.17%), yielding thinner RNFL measurements across most sectors. This signature likely reflects proprietary segmentation algorithms that position boundaries more conservatively or optical characteristics of the device’s spectral-domain design.

NIDEK exhibited positive bias (mean global bias: +10.59%), systematically overestimating RNFL thickness, particularly in temporal and nasal sectors (+17.51% and + 9.62%, respectively). This predictable overestimation could be exploited for algorithmic correction in clinical settings, yet remains unmeasured in current practice.

BMIZAR showed near-reference performance globally (mean bias: +1.93%) but exhibited sector-specific variability, particularly reduced agreement when bilateral measurements were averaged (ICC = 0.161 for inferior sector averaged data), suggesting subtle handling of interocular asymmetry.

TOPCON demonstrated the most consistent agreement with HRA across multiple analyses, with narrow LoA and low APE, suggesting algorithmic compatibility or similar segmentation principles.

These device-specific signatures are not random artifacts but reflect reproducible, platform-inherent measurement characteristics. Clinically, recognizing that each device generates a distinct measurement bias profile offers an opportunity: prospective calibration or conversion algorithms could potentially harmonize cross-platform measurements, transforming device-specific biases from sources of clinical error into quantifiable, correctable variables as recently demonstrated through machine learning-based conversion models.^[20]^

### 4.3. The repeatability–interchangeability paradox

The present study crystallizes an important methodological principle: excellent intra-device repeatability does not guarantee cross-platform interchangeability. All 5 OCT systems demonstrated outstanding short-term reproducibility (no statistically significant differences among consecutive measurements), yet substantial and clinically relevant inter-device variability persisted. This apparent contradiction has profound implications for longitudinal glaucoma monitoring and multicenter research.

### 4.4. Clinical implications and actionable recommendations

The synthesis of SDDD and the repeatability-interchangeability paradox generates several critical clinical implications that warrant specific practice modifications:

(1) **Device consistency in longitudinal monitoring:**

When glaucoma patients undergo serial RNFL imaging, consistent use of the same OCT platform is essential. Device switching introduces systematic measurement variability of 8% to 20% in nasal and temporal sectors, as demonstrated in the present healthy cohort. This concern is further supported by recent longitudinal evidence in glaucoma patients demonstrating that RNFL rates of change differ significantly between OCT platforms both globally and across clock-hour sectors, underscoring that devices cannot be used interchangeably even for progression detection.^[21]^ Whether this magnitude of variability is sufficient to simulate or mask true structural progression in glaucoma patients requires confirmation in longitudinal studies with disease populations; however, the direction and scale of the observed biases suggest this is a clinically plausible concern. Clinicians should document which device was used at each examination, and particular caution is warranted when comparing serial measurements across different platforms in the absence of platform-specific normalization.

(2) Sector-specific interpretation in multicenter settings:

In multicenter studies or practices with multiple OCT platforms, global RNFL measurements may appear concordant while sectoral measurements diverge significantly. Apparent focal RNFL loss in the nasal or temporal sector from 1 device should not be interpreted as confirmed disease progression without corroboration using the same platform or validated cross-platform algorithms. This principle is particularly critical in nasal sectors, where measurement error can exceed 20%, creating substantial risk for diagnostic misclassification.

(3) Normative database caution:

Each OCT device comes equipped with device-specific normative percentiles and reference databases. Applying absolute thickness thresholds or percentile rankings derived from 1 platform to measurements from another introduces unquantified diagnostic bias, particularly in borderline cases near disease detection thresholds (e.g., percentile 5–10 ranges where small measurement variations determine normal vs abnormal classification). Clinicians should confirm that normative comparisons match the device used for measurement.

(4) Glaucoma progression algorithms:

Automated progression detection algorithms that rely on serial RNFL measurements must account for device-dependent variables. Without platform-specific normalization, algorithms risk generating false positive alerts when multi-platform imaging is necessarily employed. Clinical software should flag device changes and either: exclude cross-platform comparisons from automated analyses; or apply documented conversion algorithms if such algorithms have been validated for the specific device pair.

It should be noted, however, that the inter-device agreement estimates reported herein are based on 38 independent participants. While this sample is sufficient to identify consistent directional biases and sector-dependent patterns, confirmation of the precise magnitude of these effects in larger, more demographically diverse cohorts would strengthen the robustness of the conclusions.

It is essential to note that all clinical recommendations above are derived from data collected in healthy eyes. While the identified device-specific bias signatures are systematic and reproducible within this normative cohort, their behavior in eyes with established glaucomatous RNFL damage, where focal thinning, asymmetric fiber bundle loss, and sub-threshold RNFL values are present, has not been assessed in this study. The clinical stakes of inter-device variability may be higher in glaucomatous eyes precisely because the signal-to-noise ratio for true structural progression detection is narrower. Future studies should prospectively evaluate whether the SDDD phenomenon and device-specific bias profiles identified here are maintained, exaggerated, or modified in eyes across the glaucoma severity spectrum.

## 5. Strength and limitations

A major strength of this study is the direct, head-to-head comparison of peripapillary RNFL thickness measurements obtained using 5 different OCT systems in the same healthy individuals during a single visit. This approach minimized biological variability and enabled a clear assessment of device-related differences under standardized imaging conditions. All scans were acquired by a single experienced operator, reducing operator-dependent variability. In addition, the inclusion of repeated measurements for each device allowed robust evaluation of short-term intra-device repeatability for both global and sectoral RNFL parameters.

Another strength is the comprehensive statistical approach, incorporating repeatability analyses, ICCs, and Bland–Altman agreement methods. This combination provided a nuanced evaluation of both reliability and agreement, allowing clinically meaningful interpretation of inter-device comparability beyond correlation-based metrics. The exclusive use of a single experienced operator for all measurements across all 5 devices and all participants eliminates interobserver variability by design, ensuring that the device-specific differences identified in this study reflect platform-inherent characteristics rather than operator-dependent variation. The focus on a healthy population further strengthens the study by offering a normative reference framework relevant to routine clinical practice.

Several limitations should be acknowledged. First and most importantly, this study exclusively included healthy volunteers, which fundamentally limits the direct applicability of our findings to glaucoma patients. In diseased eyes, RNFL architecture differs substantially from normative profiles: focal thinning, sectoral dropout, and retinal ganglion cell loss alter the tissue contrast and boundary characteristics that automated segmentation algorithms rely upon. Consequently, device-specific segmentation biases, particularly in the nasal and temporal sectors, may be amplified, attenuated, or distributed differently in eyes with glaucomatous damage compared to the healthy cohort studied here. Whether the magnitude and direction of the device-specific bias signatures identified in the present study are preserved in glaucomatous eyes remains an open and clinically critical question that requires dedicated investigation in disease populations. Until such validation is available, our findings should be interpreted as establishing a normative framework for measurement variability rather than directly quantifying the clinical risk in glaucoma monitoring scenarios.

Second, while the total number of RNFL measurements acquired was substantial owing to 3 repeated scans per eye per device (yielding up to 30 measurements per participant), the number of independent participants (n = 38) remains relatively modest. Inter-device statistical estimates, including ICC and Bland–Altman LoA, are sensitive to participant-level sample size, not measurement count. With n = 38, the 95% confidence intervals around ICC estimates are necessarily wide, and some device-pair comparisons, particularly those yielding moderate ICC values (e.g., 0.36–0.65), should be interpreted with appropriate caution, as these estimates may shift with larger cohorts. Larger independent validation studies are needed to confirm the stability and generalizability of the findings. Specifically, 2 aspects of the present results are particularly sensitive to sample size. First, the SDDD framework was characterized in a single cohort of 38 healthy participants; whether this sectoral divergence pattern replicates consistently across larger, more demographically diverse populations remains to be established. Second, the device-specific bias signatures identified here (e.g., NIDEK’s systematic positive bias, HUVITZ’s systematic negative bias) demonstrated directional consistency across multiple analytical approaches, lending internal support to their reliability; however, the precise magnitude and ranking of these signatures require confirmation in independent cohorts before they can serve as generalizable clinical reference standards for cross-platform normalization. Additionally, repeatability was assessed within a single session only, precluding conclusions regarding long-term measurement stability. Finally, while Heidelberg Spectralis was selected as the reference device based on its extensive validation record, active eye-tracking technology, widespread precedent in comparative OCT literature, and empirically lowest within-session CV% (0.42%) among the 5 devices in this study, the absence of an external gold standard for absolute RNFL thickness means that all reported biases are relative to Spectralis rather than absolute deviations from true tissue thickness.

Finally, while interobserver variability was eliminated by design through the use of a single operator, formal assessment of intraobserver variability using a dedicated test–retest protocol with a defined inter-session interval was not performed. The within-session repeated measurements used in this study provide an approximation of short-term intraobserver consistency but do not capture day-to-day or week-to-week intraobserver variability, which may be relevant in longitudinal clinical monitoring contexts.

## 6. Conclusions

The synthesis of these findings converges on a critical clinical message: RNFL measurements are not interchangeable across OCT platforms, and this limitation is disproportionately consequential in nasal and temporal sectors where measurement variability is highest. The potential risk of misinterpreting device-related measurement bias as apparent disease progression, particularly in focal, sector-specific RNFL thinning patterns, warrants attention in clinical decision-making, particularly when longitudinal OCT data are obtained from different devices. This inference, derived from a healthy population, requires prospective validation in glaucoma cohorts but is supported by the magnitude and reproducibility of the observed device-specific bias signatures. Glaucoma progression algorithms that rely on serial RNFL measurements must account for device-dependent variables or risk generating false alerts when multi-platform imaging is necessarily employed.

While intra-device repeatability is excellent, inter-device RNFL measurements remain substantially discordant, particularly in nasal and temporal sectors. In this study, this phenomenon is termed SDDD. These findings demonstrate that RNFL measurements are not merely device-dependent but sector-dependent, fundamentally limiting cross-platform interchangeability and necessitating consistent device use for longitudinal glaucoma assessment.

In conclusion, peripapillary RNFL thickness measurements obtained using different OCT systems demonstrate excellent short-term repeatability but limited inter-device agreement in healthy eyes.^[22–24]^ Systematic and sector-dependent differences between devices indicate that RNFL measurements are strongly platform-specific and should not be used interchangeably in clinical practice or research settings. While certain device pairs showed relatively higher agreement, clinically relevant bias and wide LoA persist across most comparisons. These findings underscore the importance of consistent device use for longitudinal RNFL assessment and highlight the need for caution when interpreting absolute RNFL values across different OCT platforms.

Until validated cross-platform conversion algorithms or device-harmonization strategies become available and clinically implemented, RNFL measurements should be interpreted exclusively within the context of the device used, with heightened vigilance for sector-dependent variability, particularly in nasal and temporal regions where measurement uncertainty is greatest and clinical misclassification risk is highest.

## Author contributions

**Conceptualization:** Fevzi Akkan, Ebru Görgün.

**Data curation:** Fevzi Akkan.

**Formal analysis:** Fevzi Akkan.

**Investigation:** Fevzi Akkan, Ebru Görgün.

**Methodology:** Fevzi Akkan, Ebru Görgün.

**Project administration:** Fevzi Akkan.

**Validation:** Ebru Görgün.

**Writing – original draft:** Fevzi Akkan, Ebru Görgün.

**Writing – review & editing:** Fevzi Akkan, Ebru Görgün.






