# Supplementary figures and images for: Sector-dependent device discordance of RNFL measurements: A prospective cross-sectional study of cross-platform variability and interchangeability in glaucoma monitoring
Source: Medicine (Baltimore). 2026 Jul 10;105(28):e49704. doi: 10.1097/MD.0000000000049704 (PMC13363188; doi:10.1097/MD.0000000000049704)

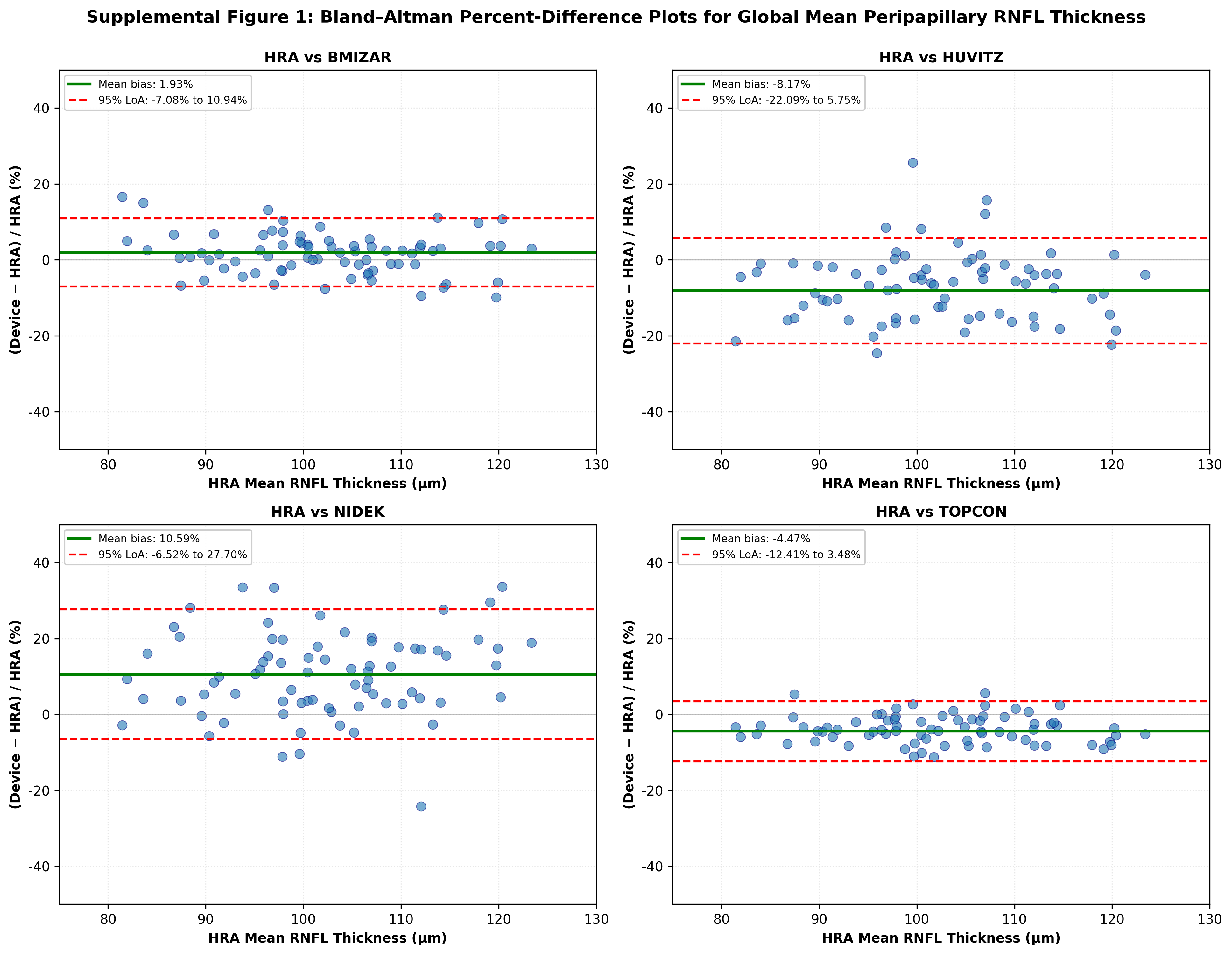

Supplement: Supplementary file 1 [file medi-105-e49704-s001.tiff]

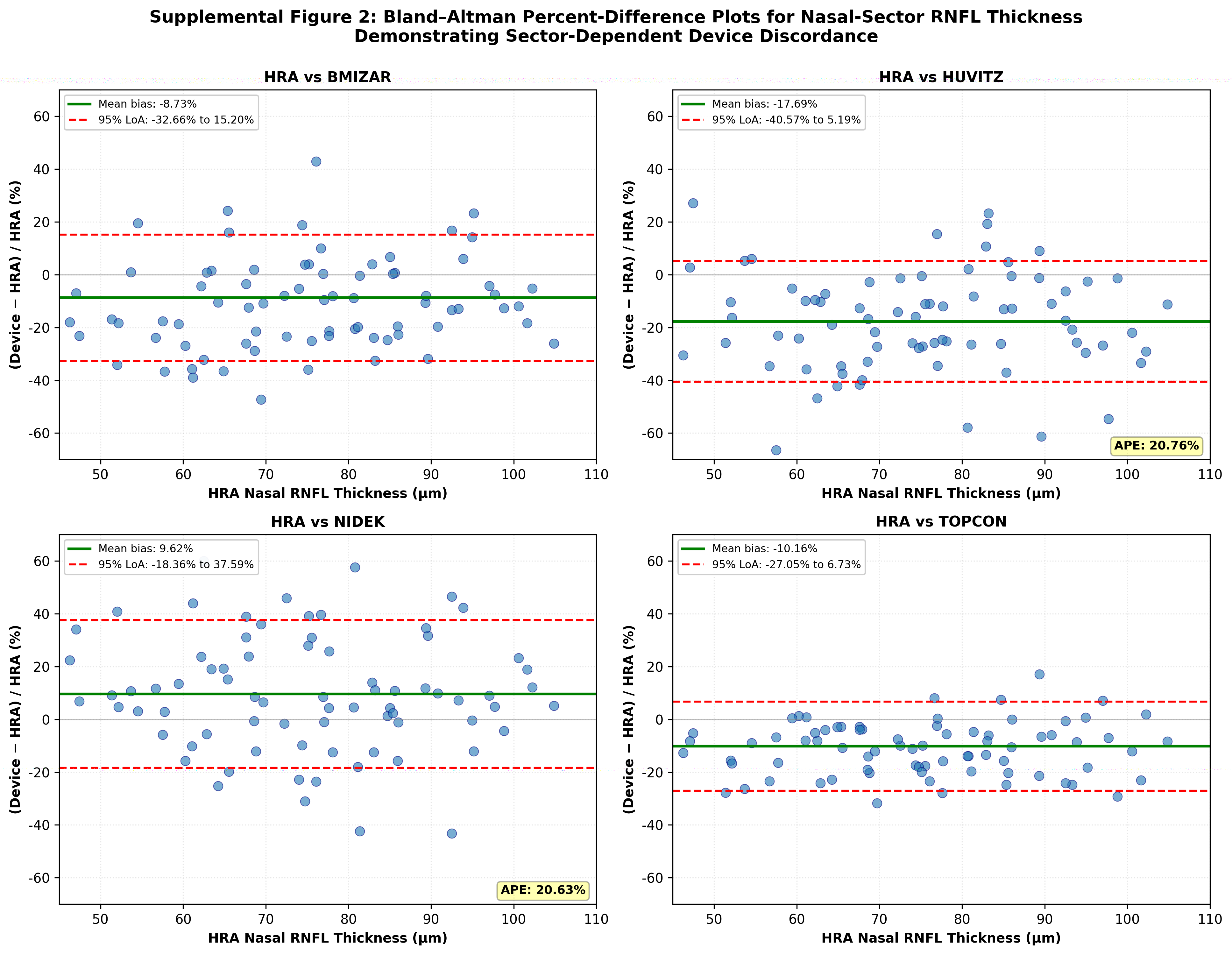

Supplement: Supplementary file 2 [file medi-105-e49704-s002.tiff]

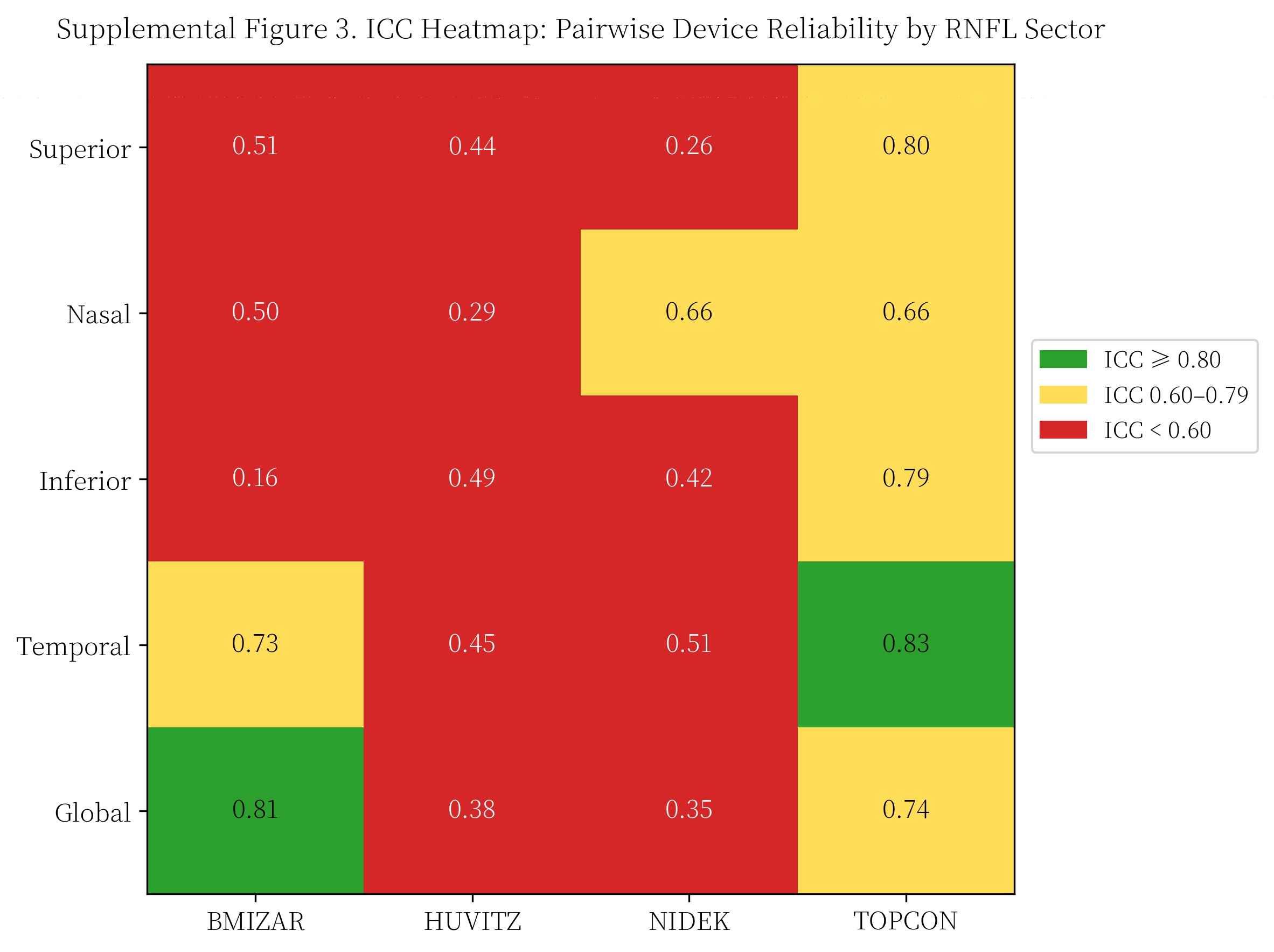

Supplement: Supplementary file 3 [file medi-105-e49704-s003.tiff]
